# The impact of ICOSL/ICOS pathway-regulated long non-coding RNAs on liver fibrosis in mice infected with *Schistosoma japonicum*

**DOI:** 10.1186/s13071-024-06399-y

**Published:** 2024-07-23

**Authors:** Ping Huang, Jing Xu, Suqin Jiang, Yanan Zhang, Xinyi Wang, Chunrong Xiong, Chaoming Xia

**Affiliations:** 1 Department of Parasitology, School of Biology & Basic Medical Sciences, Suzhou Medical College of Soochow University, Suzhou, Jiangsu Province China; 2 MOE Key Laboratory of Geriatric Diseases and Immunology, Suzhou Key Laboratory of Pathogen Bioscience and Anti-Infective Medicine, School of Biology & Basic Medical Sciences, Suzhou Medical College of Soochow University, Suzhou, Jiangsu Province China; 3 Jiangsu Institute of Parasitic Diseases, Wuxi City, Jiangsu Province China

**Keywords:** Schistosomiasis, Liver fibrosis, Hepatic stellate cells, ICOSL/ICOS, lncRNAs

## Abstract

**Background:**

The primary pathogenic mechanism of schistosomiasis-associated liver fibrosis involves the deposition of schistosome eggs, leading to the formation of liver egg granulomas and subsequent liver fibrosis. Hepatic stellate cells are abnormally activated, resulting in excessive collagen deposition and fibrosis development. While specific long non-coding RNAs (lncRNAs) have been associated with fibrotic processes, their roles in schistosomiasis-associated liver fibrosis remain unclear.

**Methods:**

Our previous research indicated that downregulating the ICOSL/ICOS could partially alleviate liver fibrosis. In this study, we established a schistosomiasis infection model in C57BL/6 and ICOSL knockout (KO) mice, and the liver pathology changes were observed at various weeks postinfection (wpi) using hematoxylin and eosin and Masson’s trichrome staining. Within the first 4 wpi, no significant liver abnormalities were observed. However, mice exhibited evident egg granulomas and fibrosis in their livers at 7 wpi. Notably, ICOSL-KO mice had significantly smaller pathological variations compared with simultaneously infected C57BL/6 mice. To investigate the impact of lncRNAs on schistosomiasis-associated liver fibrosis, quantitative real-time polymerase chain reaction (RT-qPCR) was used to monitor the dynamic changes of lncRNAs in hepatic stellate cells of infected mice.

**Results:**

The results demonstrated that lncRNA-H19, -MALAT1, -PVT1, -P21 and -GAS5 all participated in liver fibrosis formation after schistosome infection. In addition, ICOSL-KO mice exhibited significantly inhibited expression of lncRNA-H19, -MALAT1 and -PVT1 after 7 wpi. In contrast, they showed enhanced expression of lncRNA-P21 and -GAS5 compared with C57BL/6 mice, influencing liver fibrosis development. Furthermore, small interfering RNA transfection (siRNA) in JS-1 cells in vitro confirmed that lncRNA-H19, -MALAT1, and -PVT1 promoted liver fibrosis, whereas lncRNA-P21 and -GAS5 had the opposite effect on key fibrotic molecules, including α- smooth muscle actin and collagen I expression.

**Conclusions:**

This study uncovers that ICOSL/ICOS may play a role in activating hepatic stellate cells and promoting liver fibrosis in mice infected with *Schistosoma japonicum* by dynamically regulating the expression of specific lncRNAs. These findings offer potential therapeutic targets for schistosomiasis-associated liver fibrosis.

**Graphical Abstract:**

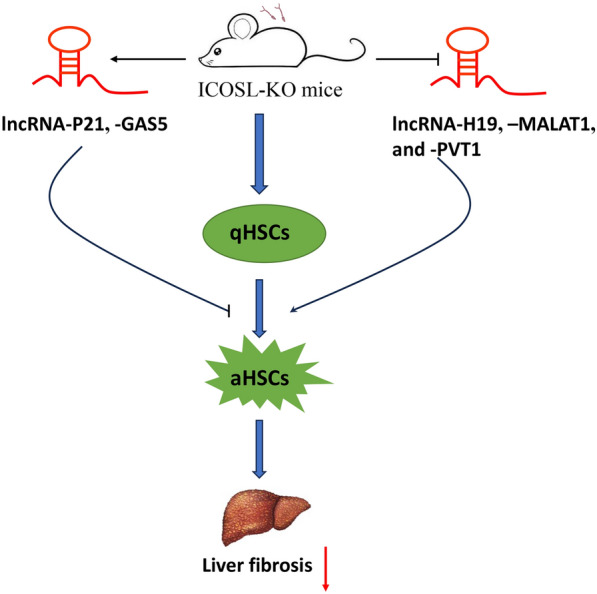

**Supplementary Information:**

The online version contains supplementary material available at 10.1186/s13071-024-06399-y.

## Background

Schistosomiasis, a severe neglected tropical disease, ranks second only to malaria among common human parasitic diseases in terms of the risk of transmission and the number of infections. Globally, schistosomiasis is endemic in 78 countries and regions, threatening approximately 779 million people, with a conservative estimate exceeding 250 million infections and 140 million symptomatic cases, and is a pressing public health issue requiring worldwide attention [1, 2]. Schistosomiasis is primarily categorized into intestinal schistosomiasis and urogenital schistosomiasis. The former is mainly caused by *Schistosoma mansoni*, *S. japonicum*, *S. mekongi*, *S. guineensis*, and related *S. intercalatum*, while urogenital schistosomiasis is predominantly caused by the species *S. haematobium* [2]. Among these, *S. mansoni* and *S. japonicum* are the most prevalent freshwater flukes of the genus *Schistosoma* that cause liver pathological alterations in humans [3, 4]. Schistosomes undergo a complex life cycle with four distinct developmental stages (cercariae, schistosomula, adults, and eggs) upon infecting definitive hosts (humans, cattle, pigs, dogs, etc.), all of which induce damage to the hosts [5]. Upon penetration of the definitive host’s skin, the cercariae transform into schistosomula, and trigger small papules at the invasion site accompanied by pruritus, known as cercarial dermatitis. After a brief stay on the skin, the schistosomula migrates with blood through the host’s veins, causing mechanical damage to organs along the way. Subsequently, they transition to the hepatic portal veins, continues to develop until the sexual organs are differentiated and mature, and migrates to the mesenteric vein and settles down to spawn. Eggs constitute the predominant pathogenic stage of schistosomes, with a single female worm capable of producing upwards of 1 million eggs throughout its entire life cycle [6]. Some eggs are excreted in feces or urine and carried out the next life cycle in a suitable environment, while others deposit in tissues such as liver and intestine, releasing soluble egg antigens (SEA) through micropores in the eggshell that provoke sustained immune responses in the host. This stimulation leads to profound immunopathological damage and progressive organ dysfunction [7].

Schistosomiasis liver fibrosis originates from granulomatous reactions to *Schistosoma* eggs, and its fundamental mechanism revolves around the activation of hepatic stellate cells (HSCs) [8]. These activated HSCs proliferate and differentiate into myofibroblasts, contributing significantly to collagen secretion and liver fibrosis development. Notably, activated HSCs release pro-fibrogenic factors like α-smooth muscle actin (α-SMA), collagen I, and collagen IV. Consequently, cytoplasmic expansion occurs, leading to substantial synthesis of proteoglycans and collagen, resulting in the excessive production of extracellular matrix (ECM). Once ECM synthesis exceeds the body's degradation capacity, excessive deposition within the liver occurs, triggering fibrogenesis [9]. Thus, inhibiting HSCs activation represents a potential therapeutic strategy for liver fibrosis.

HSCs can be activated by a variety of cytokines and cellular signal transduction pathways. Long non-coding RNAs (lncRNAs), a class of non-coding RNAs with lengths exceeding 200 nucleotides and lacking protein-coding capacity, regulate gene expression through diverse transcriptional loci in the genome [10]. They play crucial roles in cell differentiation, embryonic development, and human diseases [11–13]. Recent studies have linked specific lncRNAs with liver fibrosis. Notably, lncRNA-H19, -MALAT1, and -PVT1 are positively correlated with liver fibrosis, while lncRNA-P21 and -GAS5 are potential inhibitors of the liver fibrosis process [14, 15]. However, the mechanistic roles of different lncRNAs in host liver fibrosis induced by *Schistosoma* infection remain unclear. Inducible costimulator (ICOS) and its ligand (ICOSL) belong to CD28/B7 family, providing a second signal for T cell activation, regulating T cell differentiation and function, and promoting lymphokine secretion [16]. Our previous study showed that downregulation of ICOS signaling alleviated liver fibrosis lesions in mice infected with *S. japonicum* [17]. Hence, we aim to establish an *S. japonicum* infection model using ICOSL knock out (ICOSL-KO) and C57BL/6 wild-type mice to explore whether the ICOSL/ICOS signaling pathway modulates specific lncRNAs to participate in HSCs activation, thereby influencing the progression of liver fibrosis. This research aims to uncover the molecular mechanisms underlying schistosomiasis-associated fibrosis.

## Methods

### Animals

B6.129P2-Icosl^tm1Mak^/J (JAX no. 004657, referred to as ICOSL-KO) mice on a C57BL/6 background and their wild-type littermates were obtained from the Jackson Laboratory (Bar Harbor, ME, USA). Genotype identification was conducted when the mice reached 3 weeks of age, and male mice aged 6–8 weeks were selected for subsequent experiments. All mice were housed under specific pathogen-free conditions at the laboratory animal research facility of Soochow University. *S. japonicum-*carrying *Oncomelania hupensis* was purchased from Shanghai Municipal Center for Disease Control and Prevention (Shanghai, China) and carefully maintained in a petri dish with moist papyrus.

### Mice model

Detailed procedures involved immersing the *O. hupensis* in chlorine-free water at 25 ℃ under light for 2 h to induce cercariae release, and the released cercaria was collected for gender identification. Based on these results, 5 female and 7 male cercariae were selected using a seeding loop and placed onto glass coverslips, which were then attached to the shaved and moistened abdominal skin of mice for 15 min. At specific weeks postinfection (wpi), mice were euthanized using 2% pentobarbital anesthesia, and samples were collected for further analysis.

### Isolation and identification of primary murine HSCs

HSCs were isolated from mouse livers according to the improved procedure previously described [18]. Initially, the mouse portal vein was connected to a controllable flow rate pump, and the liver was perfused with phosphate-buffered saline (PBS) (Solarbio, Beijing, China) until it became engorged, at which point the inferior vena cava was severed. When the effluent became clear, RPMI-1640 (Gibco, California, USA) containing 0.04% collagenase I (Gibco) was used for continued perfusion (approximately 50 mL) until the liver softened. The liver was gently shred and transferred to 20 ml RPMI-1640 containing 0.08% pronase E (Solarbio), 0.08% collagenase I and 5 U/ml DNase I (Solarbio) at 39 °C, with 150 rpm oscillation digestion 15 min. Then, RPMI-1640 was added, and the cell suspension was filtered through a 70 μm membrane into a 50 ml tube and centrifuged at 4 °C, 400 g for 5 min to collect the supernatant containing non-parenchymal cells. The supernatant was then centrifuged twice with RPMI-1640. The cell pellets were resuspended in 5 ml of 15% OptiPrepTM (Axis-Shield, Oslo, Norway) and carefully overlaid with 5 ml of 11.5% OptiPrep, followed by topping up with 2 ml of RPMI-1640. The samples were centrifuged at 1400 g without brakes for 17 min to obtain purified HSCs. The isolated HSCs were cultured in Dulbecco’s modified Eagle medium (DMEM) supplemented with 15% premium fetal bovine serum (Gibco), 100 U/ml penicillin, and 100 μg/ml streptomycin, then placed in a CO_2_ incubator.

The isolated cells were stained with trypan blue to assess cell viability. The viability of the isolated cells was assessed using trypan blue staining. A mixture of 90 μl single-cell suspension and 10 μl of 0.4% trypan blue dye was prepared and mixed thoroughly. Then, 10 μl of this mixture was loaded onto a hemocytometer, and at least 200 cells were counted. Dead cells were stained blue, while live cells remained unstained. The viability of the primary HSCs was calculated as follows: Viability = (number of live cells/(number of live cells + number of dead cells)) × 100%. Freshly isolated HSCs exhibited a round shape and displayed spontaneous blue-green autofluorescence under 328 nm ultraviolet excitation due to the presence of vitamin A-containing lipid droplets in the cytoplasm. Following primary HSCs culture for 3 days, they were fixed with 4% paraformaldehyde for 60 min, followed by triple washing with PBS. Blocking was conducted using 5% BSA and 0.5% Triton (Amresco, Washington, USA) in PBS for 10 min, before incubating with GFAP antibody (Boster, Wuhan, China) overnight at 4 °C. After washing 3× with PBS, each slide was incubated with biotin-labeled goat anti-rabbit IgG secondary antibody at room temperature for 30 min. Finally, sbc-cy3 staining was performed, and representative images were observed under an inverted microscope (Olympus, Tokyo, Japan). Cells were randomly selected under the microscope from ten fields of view, and the percentage of GFAP-positive cells was calculated as follows: (GFAP-positive cells / total cells) × 100%.

### Histopathology

Mice were sacrificed at different timepoints postinfection, and their livers were isolated; each group included at least 5 mice. The livers were fixed in 4% paraformaldehyde for 4–5 days and then embedded in paraffin. Then, 4 μm-thick sections were made and stained with hematoxylin and eosin (H&E) or Masson’s trichrome staining for pathological analysis. Randomly selected liver tissue sections were used, and four individual egg granulomas or fibrotic areas were measured and analyzed per section. The areas of individual egg granulomas and liver fibrosis were measured and calculated using Image-Pro Plus (Media Cybernetics Inc., Maryland, USA) software. For fibrotic areas, the percentage was calculated by dividing the collagen area by the total area of the selected field of view. The data were then subjected to statistical analysis.

### Real-time quantitative PCR

Total RNA was extracted from HSCs and JS-1 cells using Trizol reagent (Thermo Fisher Scientific, Waltham, USA), and the concentration and purity of RNA was determined with a NanoDrop 2000c spectrophotometer (Thermo Fisher Scientific). The RNA was then reverse transcribed into complementary DNA using a reverse transcription kit, following the procedures described by our research group [18]. Expression levels of liver fibrosis-related genes, including α-SMA, collagen I, and lncRNAs, were assessed using SYBR Premix Ex Taq Kit (Takara, Tokyo, Japan), with GAPDH serving as an internal reference. Primer sequences for genes are listed in Table 1. Real-time quantitative PCR (RT-qPCR) experiments were performed with a Real-Time System (Bio-Rad, California, USA), and the relative gene expression levels were calculated using the 2^−ΔΔCt^ method.

**Table 1:** The sequences of genes

Gene	Forward primer sequence	Reverse primer sequence
lncRNA-H19	GACCAGATGGCTGCACTCTT	TCTTGTGCCAATGACCGGAA
lncRNA-MALAT1	TGCAGTGTGCCAATGTTTCG	GGCCAGCTGCAAACATTCAA
lncRNA-PVT1	CCCCTGGCAAGCAGTCTATC	GAGCATGCTGGGGACTTCAT
lncRNA-P21	CACGTCCTCCCAAGATAGCC	GACGACACAGGTGAGGAAGG
lncRNA-GAS5	TTGTGGGCCTGAAGAAGGTG	AGCCTTCATCCTCCTTTGCC
α-SMA	GTCCCAGACATCAGGGAGTAA	TCGGATACTTCAGCGTCAGGA
Collagen I	CCTGGCAAAGACGGACTCAAC	GCTGAAGTCATAACCGCCACTG
GAPDH	CAGATCCACAACGGATATATTGGG	CATGACAACTTTGGCATTGTGG

### siRNA synthesis and transfection

Specific small interfering RNA (siRNA) was synthesized by Gima Pharmaceutical Technology Co. Ltd (Shanghai, China) and the sequences are displayed in Table 2. The JS-1 cell line was cultured in DMEM containing 10% fetal bovine serum (Gibco), 100 U/ml penicillin, and 100 μg/ml streptomycin, cells were seeded at a density of 1 × 10^6^ cells/ml in six-well plates. Transfection were performed when the cells to be 60–80% confluent conducted with Lipofectamine® RNAiMAX (Life, California, USA) following the manufacturer’s protocol. Cells were collected for gene expression analysis 48 h post-transfection.

**Table 2:** The sequences of siRNAs of five lncRNA synthesized by Gima Pharmaceutical Technology Co. Ltd (Shanghai, China)

Gene	Sense	Anti-Sense
si-H19	GCAAGUGAUAGGAGGCCUUTT	AAGGCCUCCUAUCACUUGCTT
si-MALAT1	CACAGGGAAAGCGAGTGGTTGGTAA	TTACCAACCACTCGCTTTCCCTGTG
si-PVT1	GGGAUUUAGGCACUUUCAATT	UUGAAAGUGCCUAAAUCCCTT
si-P21	AAAUAAAGAUGGUGGAAUGTT	CAUUCCACCAUCUUUAUUUTT
si-GAS5	UGUACAUGGCUUUGUUCAGUU	CUGAACAAAGCCAUGUACAAG

### Statistical analysis

The data were subjected to statistical analysis using GraphPad Prism 7 software, and presented as the mean ± standard deviation (SD). Data were compared using a two-way analysis of variance (ANOVA) to assess differences between ICOSL-KO and C57BL/6 mice groups at each timepoint, with subsequent Šidák’s test, as necessary. Multiple groups were analyzed using one-way ANOVA followed by Dunnett’s test. A *P* value < 0.05 was considered statistically significant.

## Results

### Determination of the effect of ICOSL/ICOS signaling on liver pathology of mice infected with *S. japonicum*

**Fig. 1:**
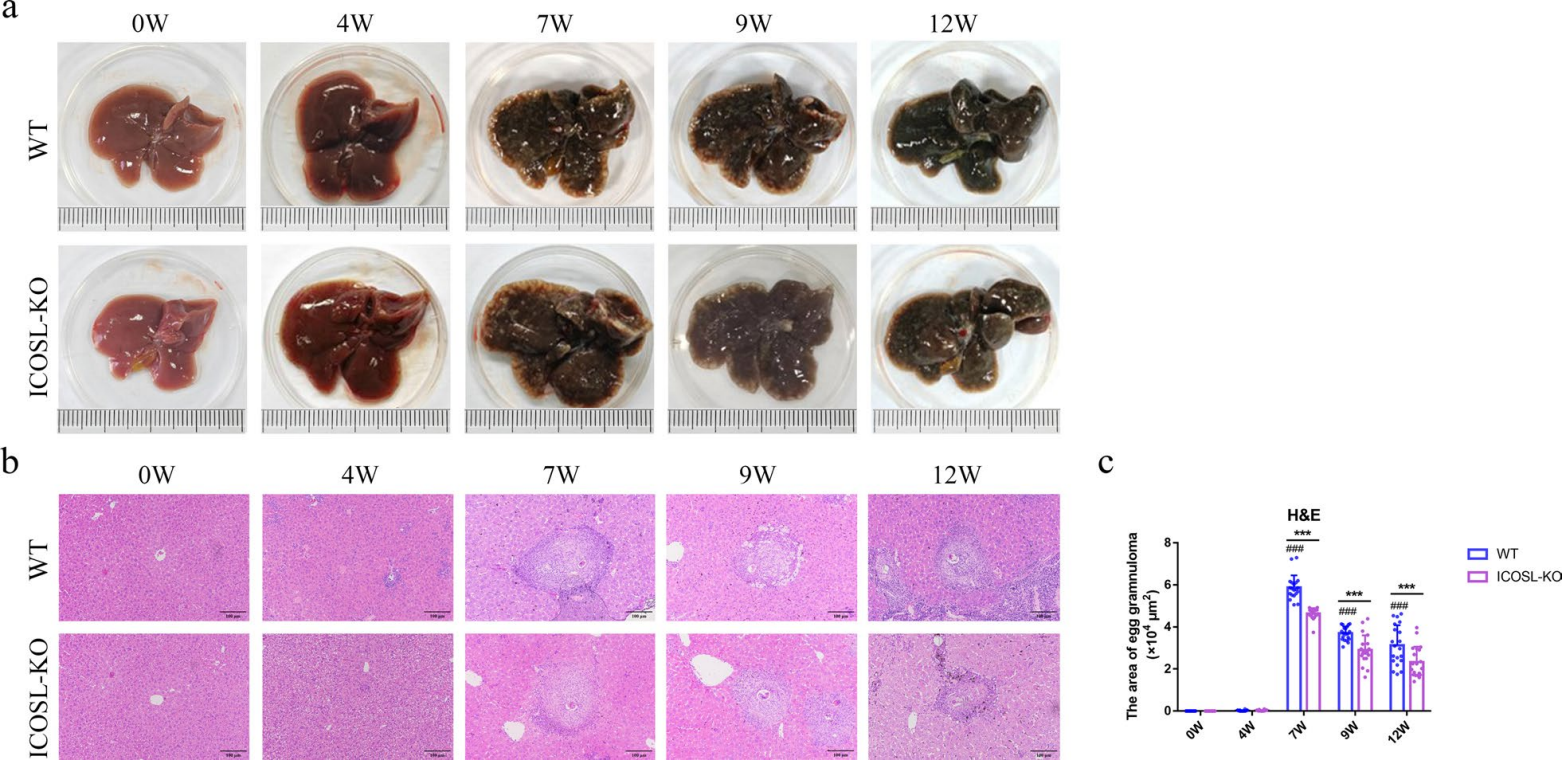
The impact of ICOSL/ICOS signaling on liver pathology in mice at different points post-infection with *S. japonicum*. **a** Anatomical photographs of the liver were captured using a camera. **b** Representative images of granulomatous lesions in paraffin-embedded liver tissues stained with H&E (scale bars, 100 μm). **c** Statistical analysis of the individual egg granuloma areas in each liver group. Data are presented as mean ± SD (*n* = 20). ^###^*P* < 0.001, compared with the 0 wpi group of C57BL/6 mice. ***P < 0.001, the inter-group comparison between ICOSL-KO and C57BL/6 mice at different wpi. WT, wild-type C57BL/6 mice; ICOSL-KO, ICOSL knockout mice

**Fig. 2:**
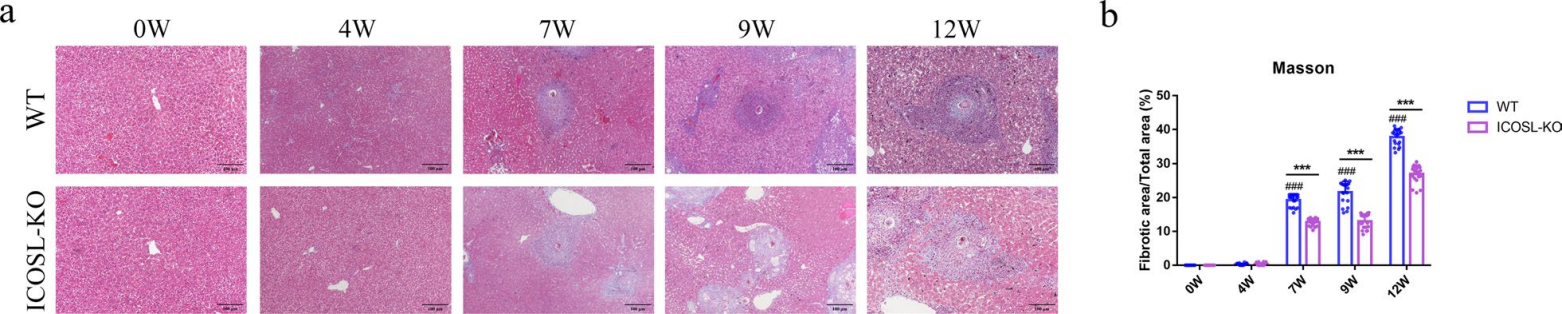
The impact of ICOSL/ICOS signaling on liver fibrosis in mice at different points postinfection with *S. japonicum*. **a** Representative images of Masson's trichrome staining depicting collagen deposition in paraffin-embedded liver tissues (scale bars, 100 μm). **b** Observation of collagen area percentage using Image-Pro Plus 6.0 software. Data are presented as mean ± SD (*n* = 20). ^###^*P* < 0.001, compared with the 0 wpi group of C57BL/6 mice. ***P < 0.001, the inter-group comparison between ICOSL-KO and C57BL/6 mice at different wpi

Freshly isolated HSCs are spherical and exhibit refractivity due to the storage of vitamin A lipid droplets, which show blue-green fluorescence under 328 nm UV excitation. After 3 days of culture, the cells increase in size, extend cellular processes, and mostly assume a spindle shape, indicating an activated state based on cell morphology. Additionally, HSCs specifically express GFAP in the cytoplasm. Immunocytochemical staining for GFAP reveals red fluorescence in the cytoplasm of HSCs (Supplementary Fig. 1). The isolated primary HSCs yielded approximately 2.5 × 10^6^ cells per mouse, with both purity and viability exceeding 90%. To accurately assess the liver pathological changes induced by *S. japonicum* in mice, we conducted pre-infection PCR identification of cercariae, with results shown in Supplementary Fig. 2. Genotype identification of C57BL/6 and ICOSL-KO mice is presented in Supplementary Fig. 3. Mouse livers were collected at different wpi (0, 4, 7, 9, and 12), at least five mice from each group were sacrificed. And the results showed that the livers of C57BL/6 mice appeared healthy, displaying a reddish hue and smooth surface during the initial 4 wpi, with no abnormalities were observed in the liver lobules and portal areas. After 7 wpi, the livers of C57BL/6 mice exhibited a grayish-brown coloration, with a granular nodular surface texture (Fig. 1a). Distinct schistosome egg granulomas were evident within the liver, surrounded by a substantial infiltration of inflammatory cells, with the granuloma area reaching its maximum size at 5.87 ± 0.58 × 10^4^ μm^2^. With the extension of infection time to 9 and 12 weeks, the granuloma area decreases to 3.69 ± 0.33 and 3.13 ± 0.95 × 10^4^ μm^2^ (*P* < 0.001), respectively, compared with 7 wpi. In addition, the liver of *S. japonicum*-infected ICOSL-KO mice also showed significant lesions from 7 wpi, but the extent of liver pathological changes was attenuated compared with C57BL/6 mice at the same period. The granuloma area of ICOSL-KO mice was 4.64 ± 0.26, 2.89 ± 0.7 and 2.33 ± 0.73 × 10^4^ μm^2^, which was significantly smaller than the infected C57BL/6 mice at 7, 9, and 12 wpi simultaneously (*P* < 0.001) (Fig. 1b, c). Masson’s trichrome results showed that the fibrotic area relative to the total area in the livers C57BL/6 mice significantly increased starting from 7 wpi (19.23% ± 1.82%), peaking at the 12 wpi (37.74% ± 2.34%) after *S. japonicum* infection. The trend of liver fibrosis change in *S. japonicum*-infected ICOSL-KO mice was consistent with that of C57BL/6 mice, there was a significant increase in collagen fiber area relative to the total area at 7 wpi (12.76% ± 1.09%), with fibrosis severity increasing with prolonged infection duration and reaching its peak at 12 wpi (26.99% ± 2.59%). However, the degree of fibrosis was significantly lower at 7, 9, and 12 wpi compared with C57BL/6 mice (*P* < 0.001) (Fig. 2).

### Detection of ICOSL/ICOS signaling on the dynamic expression of pro-fibrosis lncRNAs in primary HSCs after *S. japonicum* infection

**Fig. 3:**
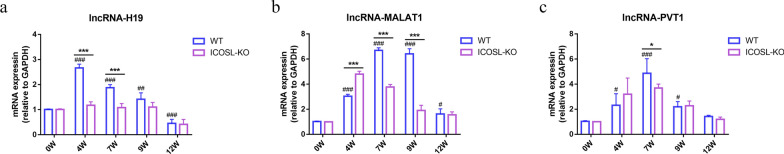
The impact of ICOSL/ICOS signaling on the dynamic expression of pro-fibrosis lncRNAs in primary HSCs following *S. japonicum* infection. **a**–**c** Relative expression of lncRNA-H19, -MALAT1, and -PVT1 were determined by RT-qPCR. Data are presented as mean ± SD (*n* = 5). ^#^*P* < 0.05, ^##^*P* < 0.01, and ^###^*P* < 0.001 compared with the 0 wpi group of C57BL/6 mice. *P < 0.05 and ***P < 0.001, the inter-group comparison between ICOSL-KO and C57BL/6 mice at different wpi

LncRNAs regulate fibrosis in human kidney, liver, and lung through various mechanisms, among which it has been found that lncRNA-H19, -MALAT1, and -PVT1 can promote fibrosis process [14, 15]. In the *S. japonicum* infection model, we found that lncRNA-H19 expression of liver HSCs in C57BL/6 mice reached a peak at the 4 wpi, and then gradually decreased. The expression levels of lncRNA-H19 increased 2.66-fold (*P* < 0.001), 1.88-fold (*P* < 0.001), and 1.42-fold (*P* < 0.01) at 4, 7, and 9 wpi, respectively, but decreased significantly at 12 wpi (*P* < 0.001), compared with 0 wpi. Interestingly, the expression levels of lncRNA-H19 in the livers of ICOS-KO mice at 4, 7, 9, and 12 wpi were 1.17-fold, 1.08-fold, 1.10-fold, and 0.42-fold, respectively. There was no significant change in expression during the first 9 wpi (*P* > 0.05), with a decrease observed at 12 wpi (*P* < 0.001), compared with 0 wpi. However, ICOSL-KO mice exhibited a significant decrease in lncRNA-19 expression at 4 and 7 wpi (*P* < 0.001), with no significant differences at other infection time points, compared with C57BL/6 mice (*P* > 0.05) (Fig. 3a). Different from the trend observed of lncRNA-19, the expression of lncRNA-MALAT increased in mouse HSCs at 4 wpi, with ICOSL-KO mice exhibiting significantly higher expression compared with C57BL/6 mice (4.79-fold and 3.03-fold, respectively, *P* < 0.001). In C57BL/6 mice, lncRNA-MALAT peaked at 7 and 9 wpi (6.69-fold and 6.42-fold, respectively), being higher than in ICOSL-KO mice (*P* < 0.001). At 12 wpi, lncRNA-MALAT1 expression decreased, but there was no significant difference in between the two mouse genotypes (Fig. 3b). Similarly, the expression of lncRNA-PVT1 in mice started to increase at 4 wpi, with C57BL/6 mice peaking at 7 wpi (4.87-fold), significantly higher than ICOSL-KO mice (3.69-fold,* P* < 0.05), followed by a gradual decrease (Fig. 3c). After infection with *S. japonicum*, C57BL/6 mice began to exhibit granuloma formation in the liver at 7 wpi, while ICOSL-KO mice showed significant alleviation of symptoms. Our results indicate that lncRNA-H19, -MALAT1, and -PVT may play a role in promoting fibrosis and are positively regulated by the ICOSL/ICOS pathway.

### Detection of ICOSL/ICOS signaling on the dynamic expression of anti-fibrosis lncRNAs in primary HSCs after *S. japonicum* infection

**Fig. 4:**
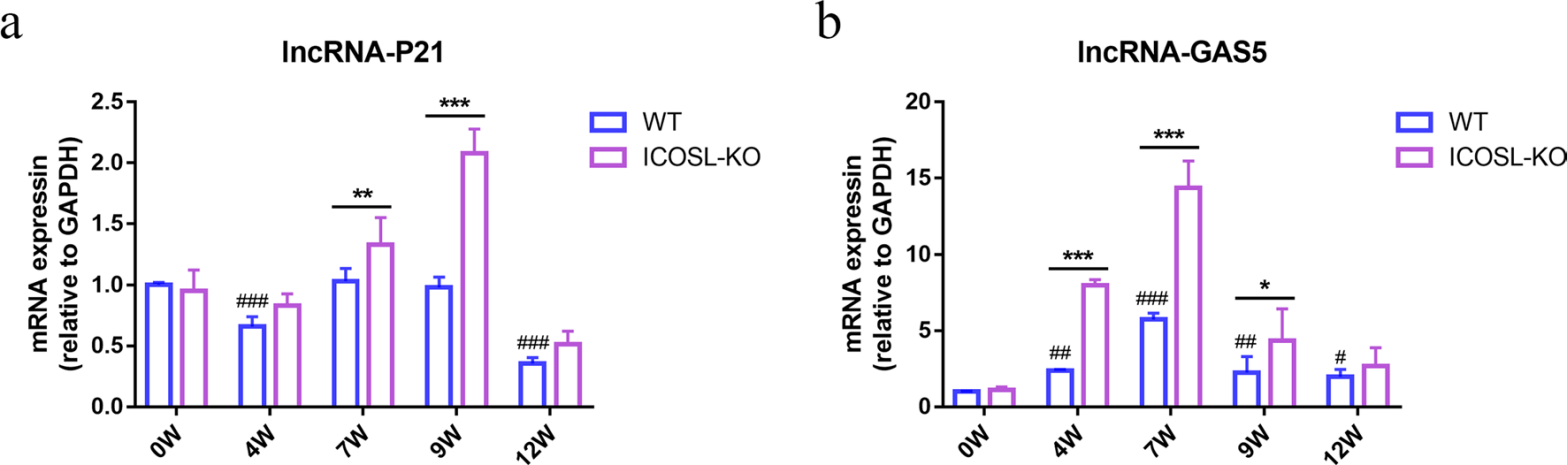
The impact of ICOSL/ICOS signaling on the dynamic expression of anti-fibrosis lncRNAs in primary HSCs following *S. japonicum* infection. **a**–**b** Relative expression of lncRNA-P21 and -GAS5 were determined by RT-qPCR. Data are presented as mean ± SD (*n* = 5). ^#^*P* < 0.05, ^##^*P* < 0.01 and ^###^*P* < 0.001 compared with the 0 wpi group of C57BL/6 mice. *P < 0.05, **P < 0.01 and ***P < 0.001, the inter-group comparison between ICOSL-KO and C57BL/6 mice at different wpi

Previous studies have suggested that lncRNA-P21 and -GAS5 may be negatively correlated with fibrotic lesions. As shown in Fig. 4, the expression of lncRNA-P21 in HSCs of C57BL/6 mice decreased at 4 and 12 wpi (*P* < 0.001), with no notable change at 7 and 9 wpi (*P* > 0.05), compared with 0 wpi. However, lncRNA-P21 in ICOSL-KO mice significantly increased at 7 wpi (1.33-fold) with *S. japonicum* infection, reaching a peak at 9 wpi (2.08-fold), and was vitally higher than in C57BL/6 mice during the same period (*P* < 0.01, *P* < 0.001). It subsequently decreased at 12 wpi, with no statistical difference compared with C57BL/6 mice (Fig. 4a). Additionly, lncRNA-GAS5 in C57BL/6 mice exhibited an initial increase at 4 wpi (2.39-fold, *P* < 0.01), reached its peak at 7 wpi (5.73-fold,* P* < 0.001), and subsequently decreased to a minimum at 12 wpi. Notably, the expression level of lncRNA-GAS5 in ICOSL-KO mice was markedly elevated compared with C57BL/6 mice at 4, 7, and 9 wpi (*P* < 0.001, *P* < 0.001 and *P* < 0.05) (Fig. 4b). These findings suggest that the ICOSL/ICOS signal may enhance the activation of HSCs and the progression of liver fibrosis by inhibiting lncRNA-P21 and -GAS5.

### Evaluation the effect of interfering with lncRNAs in liver fibrosis in vitro

**Fig. 5:**
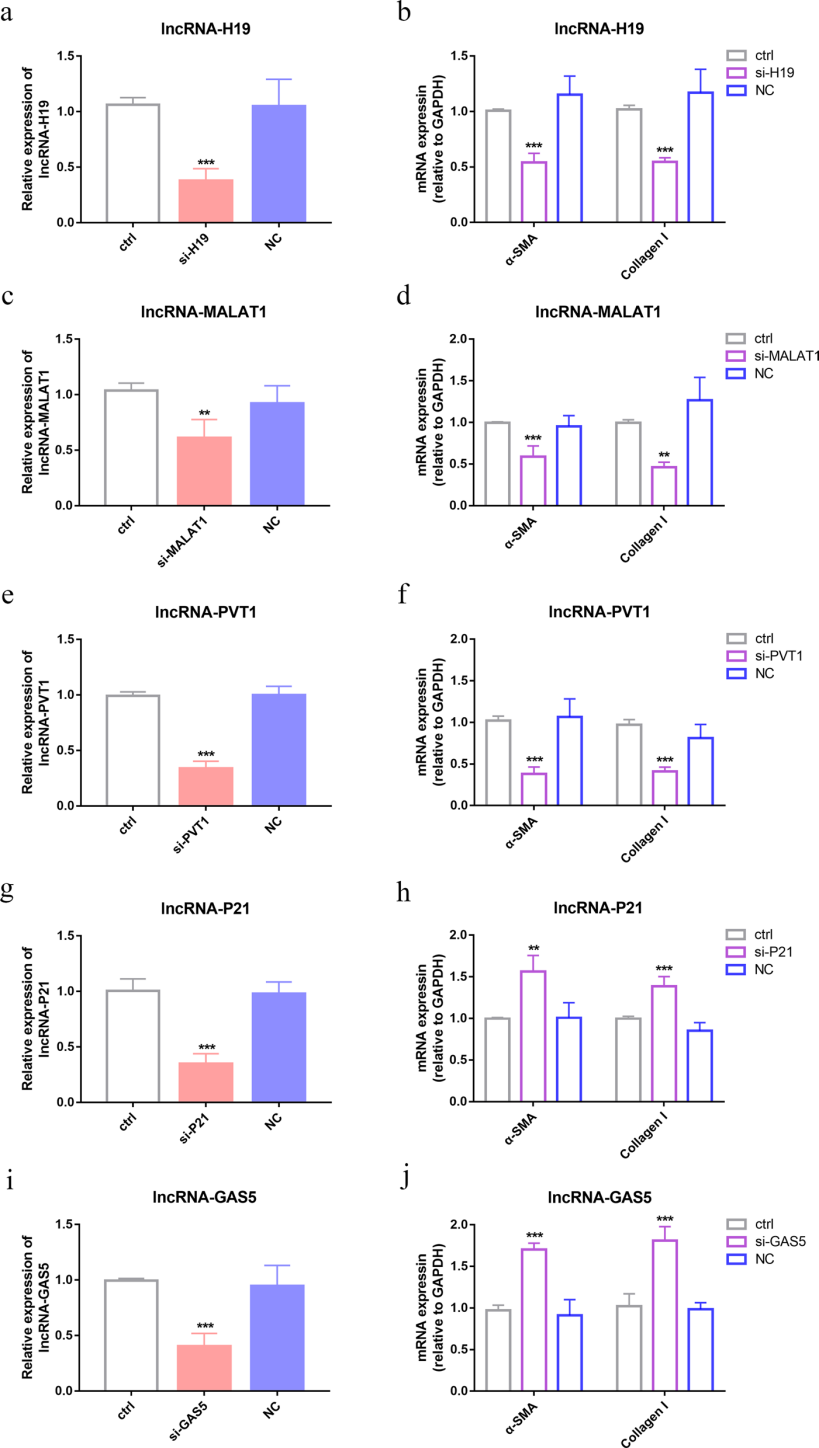
Evaluation of the effect of interfering with specific lncRNAs in liver fibrosis in vitro. **a**, **c**, **e**, **g**, **i** RT-qPCR detection of the silencing effect of different siRNAs on corresponding lncRNAs in JS-1 cells. **b**, **d**, **f**, **h**, **j** Detection of fibrosis-related molecules (α-SMA and collagen I) expression in JS-1 cells after specific siRNA silencing using RT-qPCR. Data are presented as mean ± SD (*n* = 4). *P < 0.05, **P < 0.01 and ***P < 0.001, compared with the control group. ctrl, control; NC, negative control

To further validate the role of lncRNAs in liver fibrosis, we transfected JS-1 with synthesized siRNAs and observed the impact of lncRNAs interference on the expression of pro-fibrosis molecules such as α-SMA and collagen I in vitro. The JS-1 cells were sub-cultured for 24 h, adhered to the culture dish, and differentiation 72 h later, extending pseudopodia and taking on a star-like or elongated shape, suitable for siRNA transfection (Supplementary Fig. 4). The results demonstrated that after specific siRNA transfection, the corresponding lncRNAs genes were significantly downregulated (*P* < 0.001 or *P* < 0.01) (Fig. 5). As expected, silencing lncRNA-H19, -MALAT1, and -PVT1, positively correlated with liver fibrosis, resulted in a significant decrease in α-SMA expression in JS-1 cells by approximately 46.59%, 41.02%, and 64.30% (*P* < 0.001), respectively. Similarly, collagen I expression also showed a significant reduction of 47.31%, 53.20%, and 55.71% in these conditions (*P* < 0.001 or *P* < 0.01). However, when lncRNA-P21 and -GAS5 were silenced, the expression of α-SMA increased by 1.56- and 1.71-fold (*P* < 0.01, *P* < 0.001), and the expression of collagen I also increased by 1.39- and 1.81-fold (*P* < 0.001), respectively (Fig. 5). All results indicate that ICOSL/ICOS pathway-regulated lncRNAs on liver fibrosis in mice infected with *Schistosoma japonicum*.

## Discussion

The liver plays a crucial role in maintaining normal metabolism, detoxification, and homeostasis within the body. However, when it fails to recover from various stressors, extracellular stimuli, inflammation, or injuries, it can progress to liver fibrosis [19–21]. If left untreated, liver fibrosis can lead to cirrhosis, portal hypertension, and ultimately result in liver failure and mortality. Due to its complex etiology and pathogenesis, there is currently no specific and effective treatment available. Therefore, the development of effective antifibrotic therapies is pivotal for improving patient survival.

The pathogenesis of liver fibrosis in schistosomiasis japonica is based on the continuous immune response triggered by the release of SEA from schistosome eggs deposited in the hepatic portal vein, activating HSCs and leading to granuloma fibrosis and scar tissue formation in the liver, representing an immunopathological injury [22]. Previous studies have revealed that CD4^+^ T cells play a pivotal role in the pathogenesis of *Schistosoma japonicum* infection. Among these, Th1 cells inhibit the proliferation and activation of HSCs by producing cytokines such as IFN-γ, while Th2 cells through abundant IL-13 and TGF-β production, promote fibroblasts formation and collagen synthesis. Other T cell subsets such as Th17, Tfh, and Treg cells are also involved in regulating the process of liver fibrosis [23–25]. As is well known, the activation of T cells requires dual signal stimulation. The first signal originates from the antigen, where the TCR-antigen peptide–MHC complex is delivered via CD3 to ensure the specificity of the immune response. The second activation signal, also known as costimulatory signal, is generated through the interaction between costimulatory molecules on the surface of APCs or target cells and their corresponding counterparts on T cells [26]. Common T-cell surface costimulatory molecules primarily include the immunoglobulin superfamily (such as CD28/B7-1, CTLA-4/B7-2, ICOS/ICOSL, PD-1/PD-L1, and PD-L2, etc.) and the tumor necrosis factor superfamily (including CD40/CD154, OX40/OX40L, 4-1BB/4-1BBL, etc.) [27]. The interactions among these costimulatory molecules ensure the effective initiation, appropriate effector function, and timely termination of immune responses. Interestingly, our previous research revealed that the inducible costimulatory molecule ICOSL/ICOS might be involved in the formation of liver egg granulomas and fibrosis by influencing the polarization of CD4^+^ T cells [17, 28]. To further elucidate the role of ICOSL/ICOS signaling in the process of schistosomiasis-induced liver fibrosis, we concurrently established both *S. japonicum*-infected C57BL/6 and ICOSL-KO mice and monitored the liver pathological changes at different timepoints postinfection. The results demonstrated that during the initial 4 wpi, mice exhibited no significant lesions. By the 7 wpi, the liver displayed evident egg granulomas and fibrosis, and as the infection progressed (at 9 and 12 wpi), the granuloma size gradually reduced but the fibrotic area increased. In comparison to C57BL/6 mice, ICOSL-KO infected mice showed reduced symptoms at 7–12 wpi, indicating that ICOSL/ICOS signaling can influence liver fibrotic lesions in schistosomiasis.

LncRNAs play an indispensable role in regulating the formation of liver fibrosis, particularly in HSCs activation. Acting as competitive endogenous RNAs (ceRNAs), lncRNAs share binding sites with microRNAs (miRNAs), indirectly influencing mRNA impact related to liver fibrosis, affecting post-transcriptional gene expression and epigenetic modifications [29–31]. Research has shown that lncRNA-H19, -MALAT1, and -PVT1 are pro-fibrotic factors. In diabetic patients undergoing skin wound healing, skin PDGFRα^+^ fibroblasts-derived lnc-H19 accelerates the damaged wound healing by promoting fibroblast proliferation and macrophage infiltration through inhibiting p53 activity and releasing GDF15 [32]. Furthermore, it significantly activates TGF-β expression through the H19/miR-148a/USP4 pathway, leading to notable HSCs activation and epithelial–mesenchymal transition (EMT) of hepatocytes [33]. In non-alcoholic fatty liver disease (NAFLD), lncRNA-MALAT1 regulates hepatic lipid accumulation mediated by PPARα/CD36 through the miR-206/ARNT axis, positively correlating with the severity of NAFLD [34]. In arsenite-induced liver fibrosis, lncRNA-MALAT1 facilitates miRNA-26b regulation of COL1A2 expression, thereby activating human HSCs [35]. Hypoxia, closely linked to liver fibrosis, induces autophagy via the PVT1-miR-152-ATG14 signaling pathway, contributing to HSCs activation under hypoxic conditions in primary mouse HSCs [36]. Following *S. japonicum* infection, lncRNA-H19, -MALAT1, and -PVT1 were upregulated in mouse HSCs, while their elevation was suppressed in ICOSL-KO mice. Conversely, lncRNA-P21 and -GAS5 may inhibit liver fibrosis by regulating specific miRNAs. LncRNA-P21 enhances PTEN expression by competitively binding to miR-181b, inhibiting HSCs activation [37]. In a rat model of CCL4-induced liver fibrosis, miR-23a induces liver fibrosis through the PTEN/PI3K/Akt/mTOR/snail signaling pathway, while lncRNA-GAS5 acts as a ceRNA to decrease miR-23a expression and suppressing liver fibrosis [38]. After *S. japonicum* infection, lncRNA-P21 in ICOSL-KO mice significantly increased at 7 and 9 wpi, and lncRNA-GAS5 was significantly increased from 4 to 9 wpi compared with C57BL/6 mice. This suggests that ICOSL/ICOS signaling may promote HSCs activation and liver fibrosis by reducing the expression of antifibrotic lncRNAs.

Activation of HSCs and their continuous collagen secretion are central processes in the development of liver fibrosis, α-SMA, and collagen I serve as hallmark molecules for HSCs activation and collagen fiber formation, with their expression levels directly reflecting the degree of HSCs activation [39]. Previous experiments have shown that the expression of relevant lncRNAs can be influenced by ICOSL/ICOS intervention during the course of liver fibrosis in *S. japonicum*-infected mice. To further confirm the impact of these lncRNAs on HSCs activation in mice, we performed siRNA transfections in JS-1 cells. Silencing of lncRNA-H19, -MALAT1, and -PVT1 resulted in a significant decrease in the expression of α-SMA and collagen I. Conversely, silencing of lncRNA-P21 and -GAS5 led to a significant increase in the expression of α-SMA and collagen I. These findings suggest a positive correlation between lncRNA-H19, -MALAT1, and -PVT1 with liver fibrosis, while lncRNA-P21 and -GAS5 exhibit the opposite trend. In this study, we utilized ICOSL-KO mice and primary HSCs to demonstrate that the ICOSL/ICOS signaling pathway plays a critical role in the activation of HSCs and liver fibrosis following *S. japonicum* infection by regulating specific lncRNAs. This finding was further validated by in vitro siRNA transfection experiments in JS-1 cells. However, we regret that we were unable to perform corresponding in vivo lncRNA interference experiments in ICOSL-KO mice to observe their liver fibrosis changes. Nevertheless, this limitation also highlights a direction for future research to further elucidate the mechanisms underlying schistosomiasis-associated liver fibrosis.

## Conclusions

This study reveals the dynamic role of lncRNA-H19, -MALAT1, -PVT1, -P21, and -GAS5 in HSCs activation and liver fibrosis in *S. japonicum*-infected mice, regulated by the ICOSL/ICOS signaling pathway. These findings lay a scientific groundwork for comprehending the molecular mechanisms driving liver fibrosis in *S. japonicum* infection and present potential avenues for controlling its onset and progression.

### Supplementary Information

Supplementary file 1: Figure. 1 Morphological observation of primary HSCs cultured in vitro. (a, d) HSCs freshly isolated and cultured for 3 days were observed under bright-field microscopy. (b, c) Cells were observed for intrinsic fluorescence under inverted fluorescence microscopy using 328 nm light waves. (e, f) Immunocytochemical staining detected GFAP expression in primary HSCs from *S. japonicum*-infected mice.

Supplementary file 2: Figure. 2 Gender identification of *S. japonicum* cercariae. Lanes 1–21: each number represents a *Oncomelania* *hupensis* specimen; lane 22: male control, lane 23: female control, and lane 24: 100bp DNA ladder marker.

Supplementary file 3: Figure. 3 Genotype identification of mice. Lanes 1–9, 14–17: ICOSL-KO mice; lanes 10–13: C57BL/6 mice; lanes 18–19: positive controls; lanes 20–21: negative controls; lane 22: 100-bp DNA ladder marker.

Supplementary file 4: Figure. 4 Morphological observation of JS-1 cells after 72 h of culture (×400).

## Data Availability

All data supporting the conclusions of this study are included in the article.

## Abbreviations

*S. japonicum*: *Schistosoma japonicum*
HSCs: Hepatic stellate cells
SEA: Soluble egg antigens
α-SMA: Alpha-smooth muscle actin
ECM: Extracellular matrix
lncRNAs: Long non-coding RNAs
ICOS: Inducible co-stimulator
*O. hupensis*: *Oncomelania hupensis*
H&E: Hematoxylin–eosin
RT-qPCR: Quantitative real-time PCR
mean ± SD: Mean ± standard deviation
wpi: Weeks post infection
