# Supplementary figures and images for: The impact of ICOSL/ICOS pathway-regulated long non-coding RNAs on liver fibrosis in mice infected with Schistosoma japonicum
Source: Parasit Vectors. 2024 Jul 23;17:317. doi: 10.1186/s13071-024-06399-y (PMC11267842; doi:10.1186/s13071-024-06399-y)

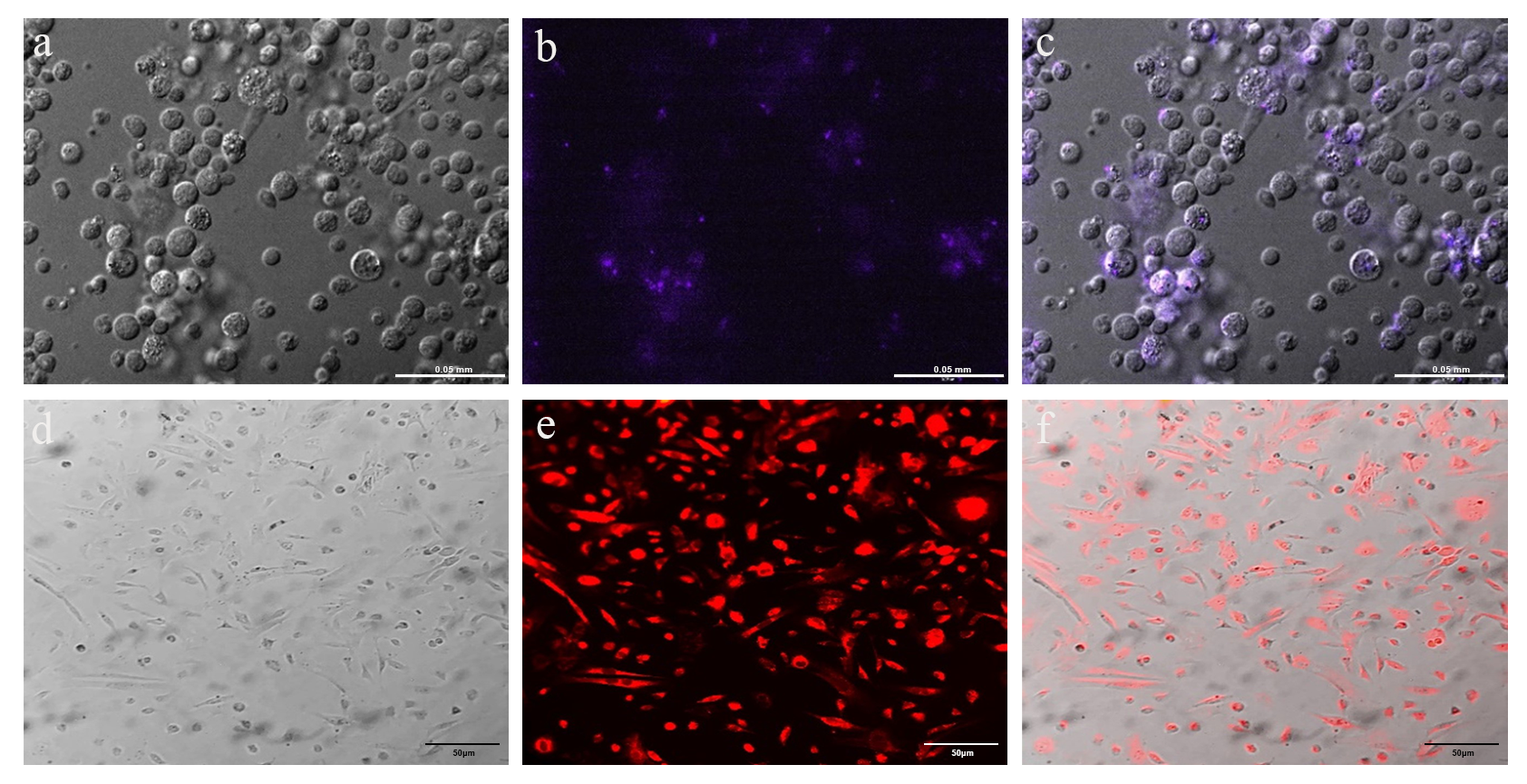

Supplement: Supplementary file 1 — Figure. 1 Morphological observation of primary HSCs cultured in vitro. (a, d) HSCs freshly isolated and cultured for 3 days were observed under bright-field microscopy. (b, c) Cells were observed for intrinsic fluorescence under inverted fluorescence microscopy using 328 nm light waves. (e, f) Immunocytochemical staining detected GFAP expression in primary HSCs from S. japonicum-infected mice. [file 13071_2024_6399_MOESM1_ESM.tif]

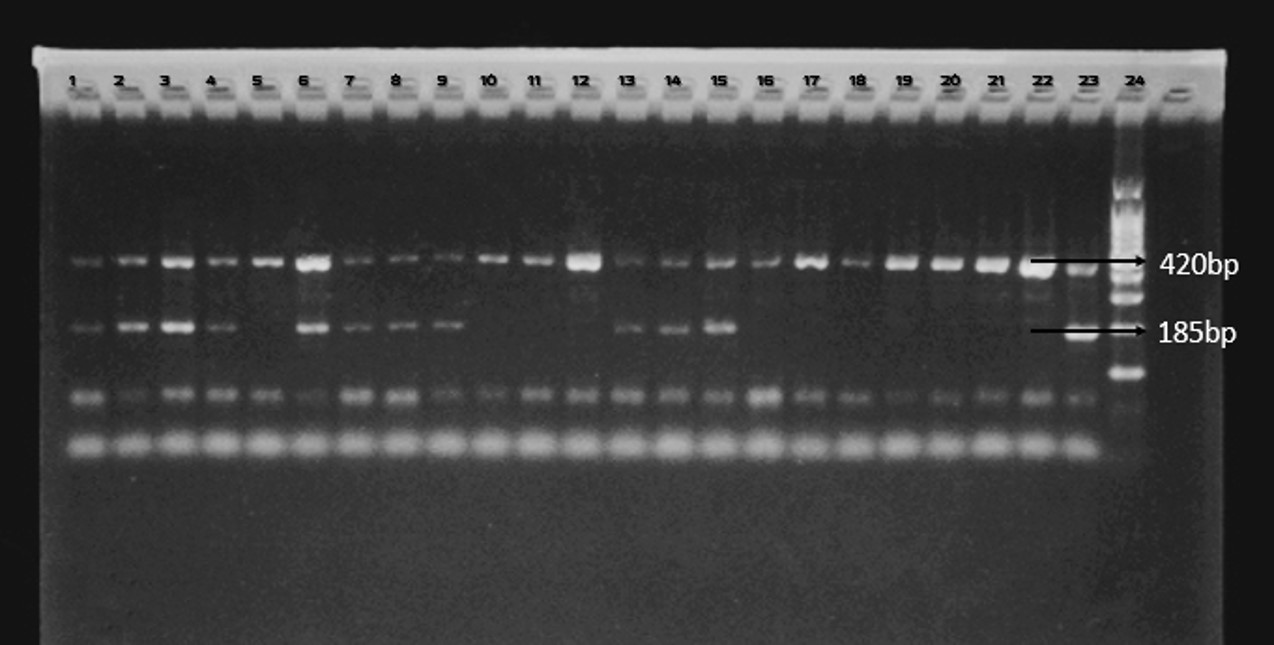

Supplement: Supplementary file 2 — Figure. 2 Gender identification of S. japonicum cercariae. Lanes 1–21: each number represents a Oncomelania hupensis specimen; lane 22: male control, lane 23: female control, and lane 24: 100bp DNA ladder marker. [file 13071_2024_6399_MOESM2_ESM.tif]

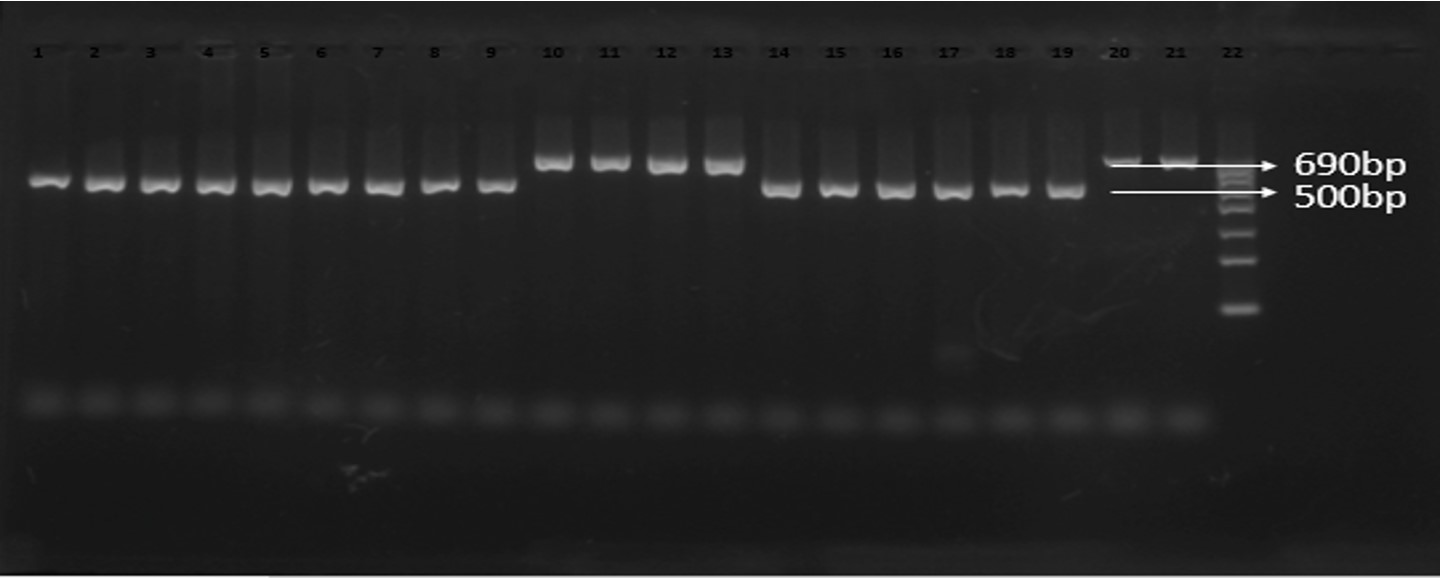

Supplement: Supplementary file 3 — Figure. 3 Genotype identification of mice. Lanes 1–9, 14–17: ICOSL-KO mice; lanes 10–13: C57BL/6 mice; lanes 18–19: positive controls; lanes 20–21: negative controls; lane 22: 100-bp DNA ladder marker. [file 13071_2024_6399_MOESM3_ESM.tif]

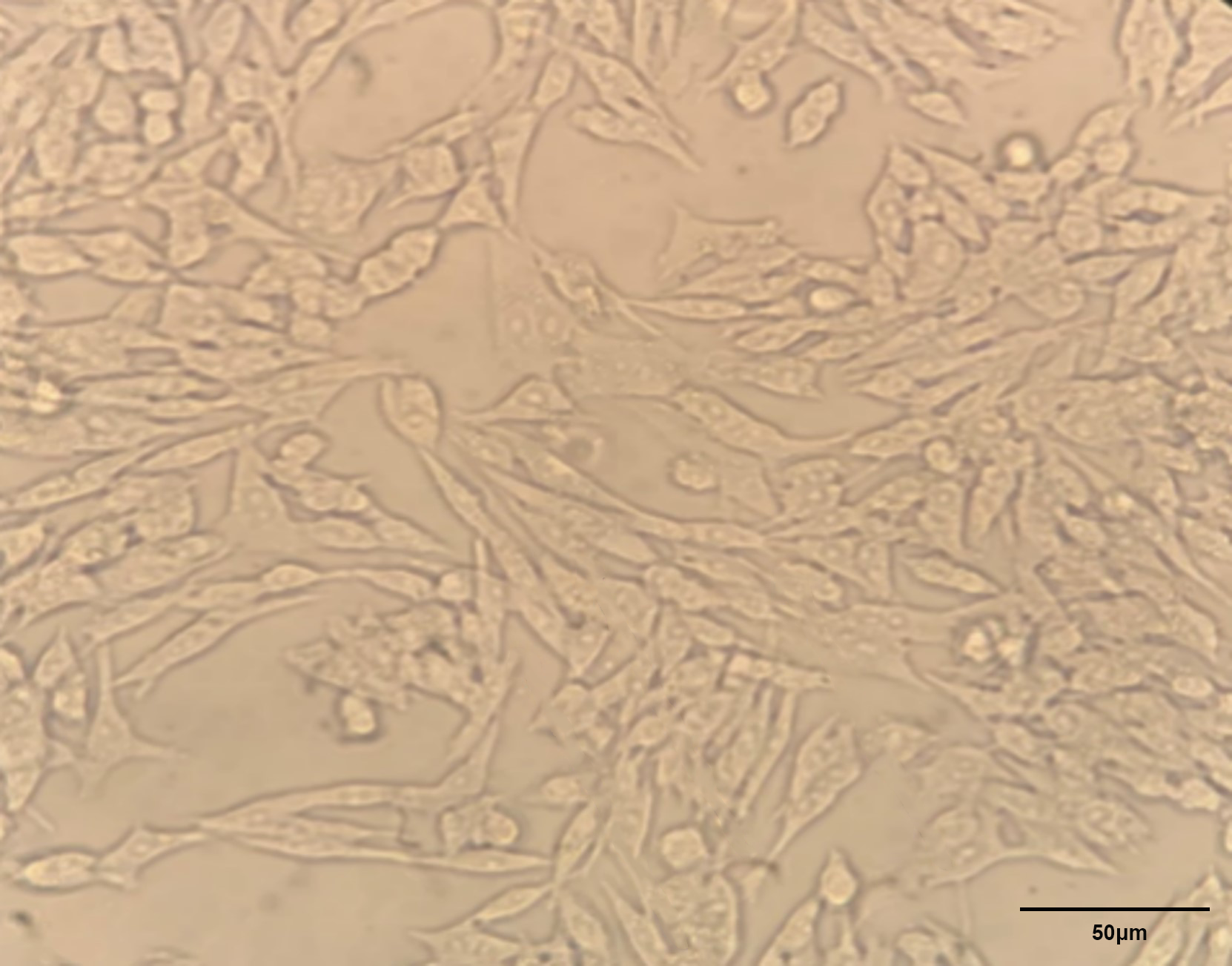

Supplement: Supplementary file 4 — Figure. 4 Morphological observation of JS-1 cells after 72 h of culture (×400). [file 13071_2024_6399_MOESM4_ESM.tif]
